# Comparing Two‐Dimensional Ellipsoid Model Variants in Estimating Three‐Dimensional Echocardiographic Right Ventricular Volume in Dogs

**DOI:** 10.1111/jvim.70232

**Published:** 2025-08-27

**Authors:** Robert Ciardullo, Brittany E. Tagg, Elisabeth Nesta, Shana B. Mintz, Romain Pariaut, Weihow Hsue

**Affiliations:** ^1^ Department of Clinical Sciences, College of Veterinary Medicine Cornell University Ithaca New York USA

**Keywords:** 4D auto RVQ, healthy, linear, pulmonary hypertension, pulmonary valve stenosis, tricuspid valve dysplasia

## Abstract

**Background:**

Determining right ventricular (RV) volume typically requires three‐dimensional imaging due to its complex shape. The ellipsoid model offers a two‐dimensional alternative, employing area‐ or linear‐based formulas with further variations depending on the echocardiographic views used for measurements.

**Hypothesis/Objectives:**

To identify which ellipsoid model variant best agrees with real‐time three‐dimensional echocardiography (RT3D) as a reference standard and to assess within‐day reproducibility.

**Animals:**

Sixty‐seven client‐owned dogs (23 normal, 44 with right‐sided heart diseases) underwent echocardiograms, with 20 normal dogs receiving a repeat examination.

**Methods:**

Prospective method comparison study. Body weight‐indexed end‐diastolic (iEDV) and end‐systolic volumes (iESV) were calculated across eight ellipsoid model variants. Agreement was assessed using concordance correlation coefficients (*r*_c_) and Bland–Altman analysis, while within‐day reproducibility was evaluated using intraclass correlation coefficients (ICC) and reproducibility coefficients.

**Results:**

The area‐ and linear‐based variants using RV parameters from the left apical four‐chamber view and perpendicular diameter from the right parasternal short‐axis view (AEM_A4C‐RPS_ and LEM_A4C‐RPS_, respectively) were the only methods to achieve moderate agreement with RT3D (*r*_c_ > 0.90). The AEM_A4C‐RPS_ showed no significant systematic bias for iEDV (median of the differences [95% confidence interval]: 0.09 [0.00–0.13]), while LEM_A4C‐RPS_ exhibited no significant systematic bias for iEDV (0.03 [−0.02–0.08]) and iESV (0.04 [−0.01–0.12]), though biases increased at larger volumes. Both methods demonstrated good reproducibility (ICC > 0.75), with iESV reproducibility significantly greater than that of methods using RV parameters from the right parasternal long‐axis four‐chamber view.

**Conclusions and Clinical Importance:**

The AEM_A4C‐RPS_ and LEM_A4C‐RPS_ offer practical RV volume estimates.

**Abbreviations**
2D: two‐dimensional
AEM: area‐based ellipsoid model
AEM_A4C‐A2C_
: area‐based ellipsoid model using the left apical four‐chamber and left apical two‐chamber views
AEM_A4C‐RPS_
: area‐based ellipsoid model using the left apical four‐chamber and right parasternal short‐axis basilar views
AEM_RPL‐A2C_
: area‐based ellipsoid model using the right parasternal long‐axis four‐chamber and left apical two‐chamber views
AEM_RPL‐RPS_
: area‐based ellipsoid model using the right parasternal long‐axis four‐chamber and right parasternal short‐axis basilar views
iEDV: body weight‐indexed end‐diastolic volume
iESV: body weight‐indexed end‐systolic volume
LEM: linear‐based ellipsoid model
LEM_A4C‐A2C_
: linear‐based ellipsoid model using the left apical four‐chamber and left apical two‐chamber views
LEM_A4C‐RPS_
: linear‐based ellipsoid model using the left apical four‐chamber and right parasternal short‐axis basilar views
LEM_RPL‐A2C_
: linear‐based ellipsoid model using the right parasternal long‐axis four‐chamber and left apical two‐chamber views
LEM_RPL‐RPS_
: linear‐based ellipsoid model using the right parasternal long‐axis four‐chamber and right parasternal short‐axis basilar views
MRI: magnetic resonance imaging
RT3D: real‐time three‐dimensional echocardiography
RV: right ventricle

## 1 Introduction

Quantitative assessment of the right heart is not routinely performed or is focused on systolic function [1]. However, abnormal right ventricular (RV) end‐diastolic and/or end‐systolic chamber size can be present in both right‐sided cardiac diseases, such as pulmonary valve stenosis [2], pulmonary hypertension [3], and tricuspid valve dysplasia [4], and left‐sided diseases, including myxomatous mitral valve disease [5] and dilated cardiomyopathy [6]. Therefore, evaluation of RV size should be included in every echocardiographic study.

Unlike the left ventricle, the RV has a crescentic shape with distinct inflow and outflow regions that are less suited for simple geometric modeling [7]. As such, while echocardiographic linear dimensions and right heart ratios are described, quantification of RV volume using two‐dimensional (2D) methods remains challenging [8, 9]. The Simpson's method of discs, a 2D technique commonly used to assess left ventricular volume, has been applied to the RV in dogs, but it severely underestimates and correlates poorly with volumes derived from advanced imaging modalities such as cardiac magnetic resonance imaging (MRI) [10]. This is likely because the formula models the chamber as a series of cylindrical disks, which incorrectly represent the RV's true shape. In contrast, three‐dimensional imaging modalities provide direct visualization of the entire RV, enabling precise delineation of the borders from all perspectives [7, 11, 12]. Cardiac MRI is considered the most accurate volumetric method in humans but requires anesthesia in dogs, which could reduce cardiac function [8, 13]. Real‐time three‐dimensional echocardiography (RT3D), which correlates well with MRI, can be comfortably performed in awake dogs with severe cardiac disease [11]. However, like MRI, it is hindered by accessibility, expertise, and costs.

**FIGURE 1:**
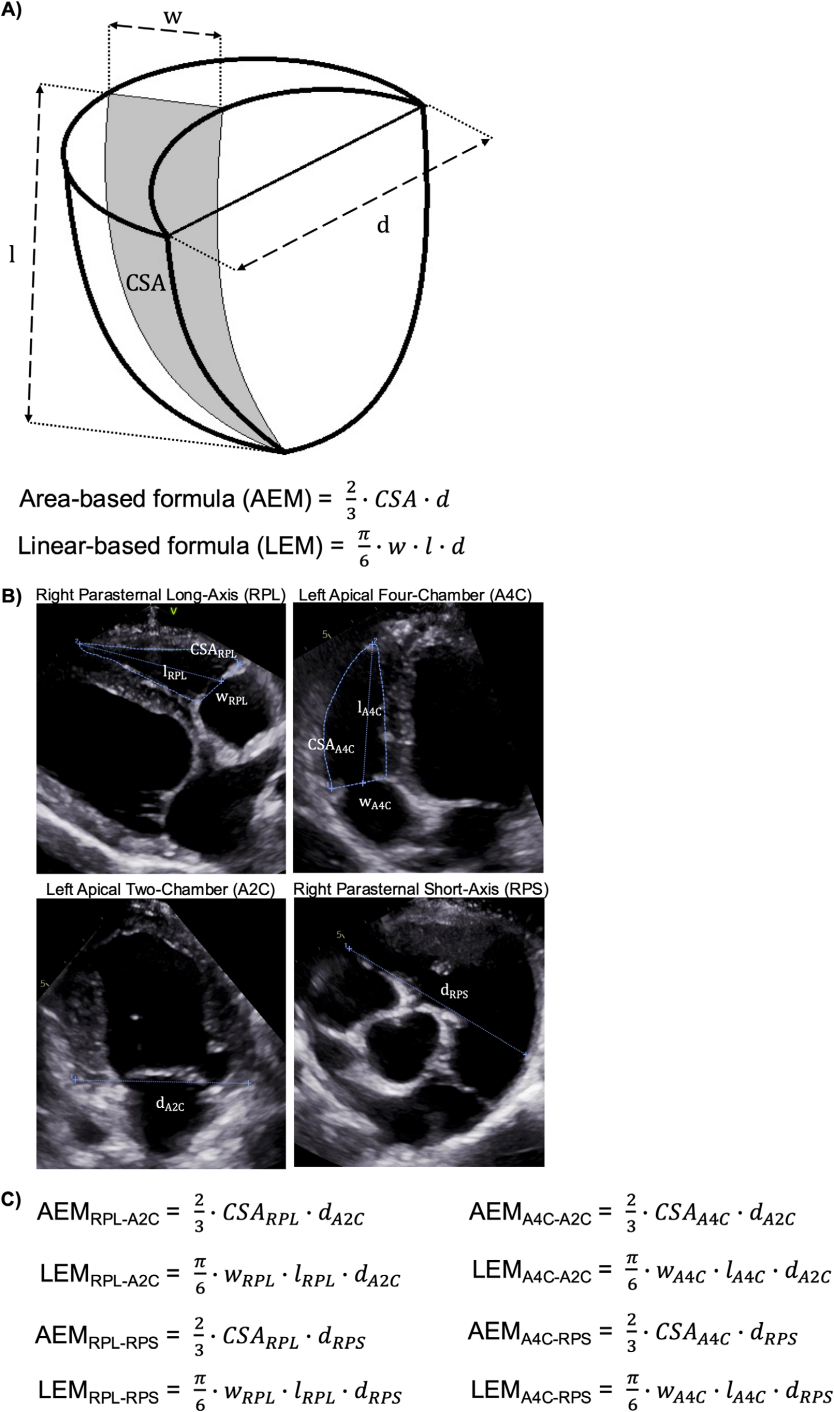
Area‐based ellipsoidal model (AEM) and linear‐based ellipsoid model (LEM) formulas. (A) The figure illustrates the key formula components, including the RV cross‐sectional area (CSA), the RV basilar width (w), the RV length (l), and the perpendicular diameter (d). (B) The RV area, basilar width, and length can be obtained on either the right parasternal long‐axis four‐chamber (RPL) or left apical four‐chamber (A4C) view, while the perpendicular diameter can be obtained on either the left apical two‐chamber (A2C) or right parasternal short‐axis basilar (RPS) view. (C) Formulas of all possible AEM and LEM variants are listed.

An echocardiographic 2D area‐based ellipsoid model (AEM) that approximates RV shape has been investigated in humans. Volume is calculated by multiplying the long‐axis cross‐sectional area of the RV (“CSA” in Figure 1A) by a perpendicular diameter (“d” in Figure 1A) and applying a geometric coefficient [14, 15]. An alternative linear‐based ellipsoid model (LEM) has also been proposed, substituting the RV area with the minor‐axis basilar width (“w” in Figure 1A) and the major‐axis length (“l” in Figure 1A) while using a different coefficient [16]. Further variations exist, as these measurements can be obtained from different echocardiographic views (Figure 1B). For example, the long‐axis RV plane can be visualized from the right parasternal or left apical four‐chamber view [9]. Similarly, the perpendicular diameter can be measured from the right parasternal short‐axis basilar view as the transverse distance between the RV lateral wall near the tricuspid annulus and the right ventricular outflow tract beneath the pulmonary valve [15], or from the left apical two‐chamber view as the maximum outer basilar diameter of the left ventricle [16]. These variations have not been systematically compared against a reference standard in dogs.

The primary objective of this study was to compare the bias and agreement of end‐diastolic and end‐systolic RV volumes calculated using all AEM and LEM variants, with RT3D as the reference standard, in healthy dogs and dogs with right‐sided heart disease. A secondary objective was to assess within‐day reproducibility of these methods in healthy dogs.

## 2 Materials and Methods

All procedures were approved by the Institutional Animal Care and Use Committee at Cornell University (protocol #: 2022‐0204). All dog owners provided written, informed consent before enrollment.

### 2.1 Animals

This single‐center, prospective method comparison study was conducted between January 2023 and November 2024. Healthy dogs were recruited from students and staff from the Cornell University College of Veterinary Medicine, while dogs with right‐sided heart disease were recruited from client‐owned dogs presenting as clinical patients to the Cornell University Hospital for Animals. Inclusion criteria for the healthy group required dogs to be free of clinical signs, have a normal physical exam (a physiologic left basilar systolic murmur graded ≤ III/VI was accepted), not be receiving any medications other than supplements, and have a normal echocardiographic exam. The echocardiographic exam was considered normal if no cardiac abnormalities were identified, left heart dimensions were within reference ranges [17], and right heart ratios were appropriate [9]. Trivial atrioventricular or semilunar valve regurgitation was permitted if inaudible on auscultation and unaccompanied by structural valve abnormalities. Inclusion criteria for the right‐sided heart disease group required a confirmed diagnosis of right‐sided heart disease without evidence of left‐sided heart enlargement based on established references ranges [17]. Eligible conditions included: pulmonary valve stenosis (pulmonary valve thickening, immobility, and/or commissural fusion with a peak pulmonary outflow velocity > 3 m/s); pre‐capillary pulmonary hypertension (at least of intermediate probability based on the American College of Veterinary Internal Medicine consensus statement [18]); tricuspid valve dysplasia (morphologically abnormal tricuspid valve, such as septal leaflet tethering or abnormal chordal attachments, with nontrivial tricuspid regurgitation on Color Doppler, first diagnosed at ≤ 2 year of age); and tricuspid valve degeneration (tricuspid valve thickening and/or prolapse with nontrivial tricuspid regurgitation exceeding mitral regurgitation in severity on Color Doppler, first diagnosed at ≥ 6 years of age). Exclusion criteria for both groups included the presence of significant arrhythmias (i.e., more than intermittent single premature complexes) or the need for sedation during imaging.

### 2.2 Experimental Design

All dogs underwent echocardiographic examination performed by a board‐certified cardiologist (W.H.). When available, a resident‐in‐training (R.C.) also conducted a separate echocardiographic exam, with operator order randomly determined by coin toss. The study was initially designed to assess interoperator reproducibility between the cardiologist and the resident. However, given the challenges of acquiring optimal RV views and the steep learning curve associated with RT3D, the protocol was modified so that all measurements were performed by the resident in conjunction with the cardiologist. Consequently, only one exam per dog was analyzed; when two exams were available, the operators jointly selected the one with the highest perceived quality before measuring. The operators were blinded to the ellipsoid model results during RT3D measurements.

If permitted, dogs within the healthy group underwent an additional echocardiographic exam at least 3 h after the initial exam. This interval was chosen to balance adequate separation—minimizing recall of prior image acquisition by the sonographer—with logistical practicality, allowing imaging to be completed in a single visit. The cardiologist alone repeated measurements on this subset while blinded to the previous results to assess within‐day reproducibility.

### 2.3 Echocardiographic Examination and Measurements

Routine M‐mode, 2D, and Doppler imaging were performed using a Vivid E95 ultrasound unit (GE Healthcare, Chicago, Illinois) equipped with several phased‐array transducers (5–12 MHz), selected based on the size of the dog, along with a 4Vc‐4D transducer for acquiring RT3D images. Simultaneous electrocardiographic monitoring was employed. Recommended tomographic imaging planes were acquired, including the right parasternal long‐axis four‐chamber view, the right parasternal short‐axis basilar view with emphasis on simultaneous visualization of the tricuspid and pulmonary valves, the left apical four‐chamber view, and the left apical two‐chamber view [19]. The four‐chamber views were focused on maximum visualization of the RV, including the apex, while minimizing foreshortening and excluding the aorta and caval structures [20].

Measurements were performed offline using dedicated software (EchoPac, GE Healthcare, Chicago, Illinois). Both end‐diastolic and end‐systolic volumes were assessed, with end‐diastole defined as the frame immediately following tricuspid valve closure and end‐systole as the frame showing the smallest RV cavity immediately before tricuspid valve opening. All measurements were averaged over three to five cycles, allowing the operators to include additional cycles if motion, respiratory artifacts, or sinus arrhythmia affected image consistency. These cycles were typically consecutive, except for RT3D, which did not allow consecutive measurements. All volumetric measurements were divided by body weight to derive indexed end‐diastolic volumes (iEDV) and indexed end‐systolic volumes (iESV).

The RV area, basilar width, and length were measured on the right parasternal long‐axis and left apical four‐chamber views as previously described (Figure 1B) [8, 9, 20]. Briefly, the RV area was derived by manually tracing the RV endocardial border, including the papillary muscles and moderator band within the cavity, with a straight line from hinge point to hinge point across the tricuspid annulus defining the basilar border of the RV. The RV basilar width was defined as the maximum distance parallel to the tricuspid annulus within the basilar third of the RV cavity. The RV length was measured as the distance from the midpoint of the tricuspid annulus to the apex. The perpendicular diameter was measured on the right parasternal short‐axis basilar and left apical two‐chamber views (Figure 1B). In the former view, the diameter spanned from the lateral tricuspid hinge point to the endocardial border of the right ventricular outflow tract beneath the pulmonary valve, crossing near the top of the aortic valve [15]. A slight clockwise rotation from the traditional right parasternal short‐axis basilar view may improve simultaneous visualization of both valves. In the latter view, the diameter spanned from epicardium to epicardium across the mitral annulus [16].

Four sets of the ellipsoid model were evaluated, each including an area‐based and a linear‐based formula (Figure 1C). The AEM was defined as $\frac{2}{3}\times \left(\mathrm{RV}\;\text{area}\right)\times \left(\text{perpendicular diameter}\right)$, while the LEM was $\frac{\pi }{6}\times \left(\mathrm{RV}\;\text{basilar width}\right)\times \left(\mathrm{RV}\;\text{length}\right)\times \left(\text{perpendicular diameter}\right)$ [14, 15, 16]. The first set derived the RV parameters, consisting of RV area, RV basilar width, and RV length, from the right parasternal long‐axis four‐chamber view and the perpendicular diameter from the left apical two‐chamber view (i.e., AEM_RPL‐A2C_ and the LEM_RPL‐A2C_). The second set derived the RV parameters from the right parasternal long‐axis four‐chamber view and the perpendicular diameter from the right parasternal short‐axis basilar view (AEM_RPL‐RPS_ and LEM_RPL‐RPS_). The third set derived the RV parameters from the left apical four‐chamber view and the perpendicular diameter from the left apical two‐chamber view (AEM_A4C‐A2C_ and LEM_A4C‐A2C_). The fourth set derived the RV parameters from the left apical four‐chamber view and the perpendicular diameter from the right parasternal short‐axis basilar view (AEM_A4C‐RPS_ and LEM_A4C‐RPS_).

Real‐time multiplane views of the RV, comprising three long‐axis views and nine short‐axis views, were obtained with multi‐beat acquisition at a target frame rate of > 30 frames per second (Figure S1). The RT3D volumes were calculated using the semi‐automated 4D Auto RVQ volume quantification module (Video S1) [21]. In the initial screen, a vertical axis was manually aligned through the tricuspid valve center and the RV apex. On the subsequent screen, the tricuspid annulus and RV apex were manually identified on the provided long‐axis view, while the anterior, lateral, and posterior points were manually identified on the provided short‐axis view. The automated endocardial border tracing was then displayed in a 3 × 3 multislice interface: the first column allowed browsing through the entire cardiac cycle; the second column showed only end‐diastolic views, and the third showed only end‐systolic views. The multislice model was reviewed in all planes to ensure accurate endocardial tracing, with manual corrections applied as needed. A time‐volume curve and various analytical values of RV volume and function were then generated. The timing of end‐diastole and end‐systole was verified before recording the volumes.

### 2.4 Statistical Analysis

All statistical analyses were performed using MedCalc (MedCalc Software Ltd., Ostend, Belgium). Normality was assessed with the Shapiro–Wilk test. Quantitative data were expressed as median (interquartile range) unless otherwise specified. *Bias* was defined as a non‐zero median of the differences between two methods, while agreement, defined as the degree of consensus between two sets of measurements, was evaluated using Lin's concordance correlation coefficient [22, 23]. The concordance correlation coefficient accounts for both the Pearson correlation coefficient, a measure of the deviation from the best‐fit line, and a bias correction factor, a measure of the deviation of the best‐fit line from the 45° line through the origin. Values of < 0.90 indicated poor agreement; 0.90–0.95 indicated moderate agreement; 0.95–0.99 indicated substantial agreement; and > 0.99 indicated almost perfect agreement [23]. In addition, Bland–Altman plots were generated to visualize the line of equality, regression line of differences, and limits of agreement [24]. Significance was set at two‐sided α = 0.05.

Within‐day reproducibility was evaluated using coefficients of variation (which quantify the differences relative to the mean), intraclass correlation coefficients (which quantify the association between different sessions), and 95% reproducibility coefficients (which quantify the limits between which 95% of repeated measures are expected to fall, expressed in the same units as the measurement of interest) [25]. Coefficients of variation were derived using the within‐subject standard deviation and were calculated as: (within‐subject standard deviation ÷ overall mean) × 100. The intraclass correlation coefficients were calculated using a two‐way model with absolute agreement and were reported by single measures; values < 0.5 indicated poor reproducibility, 0.5–0.75 indicated moderate reproducibility, 0.75–0.90 indicated good reproducibility, and > 0.90 indicated excellent reproducibility [26]. The 95% reproducibility coefficients were calculated as 1.96 × standard deviation of the differences between two sets of measurements.

## 3 Results

**FIGURE 2:**
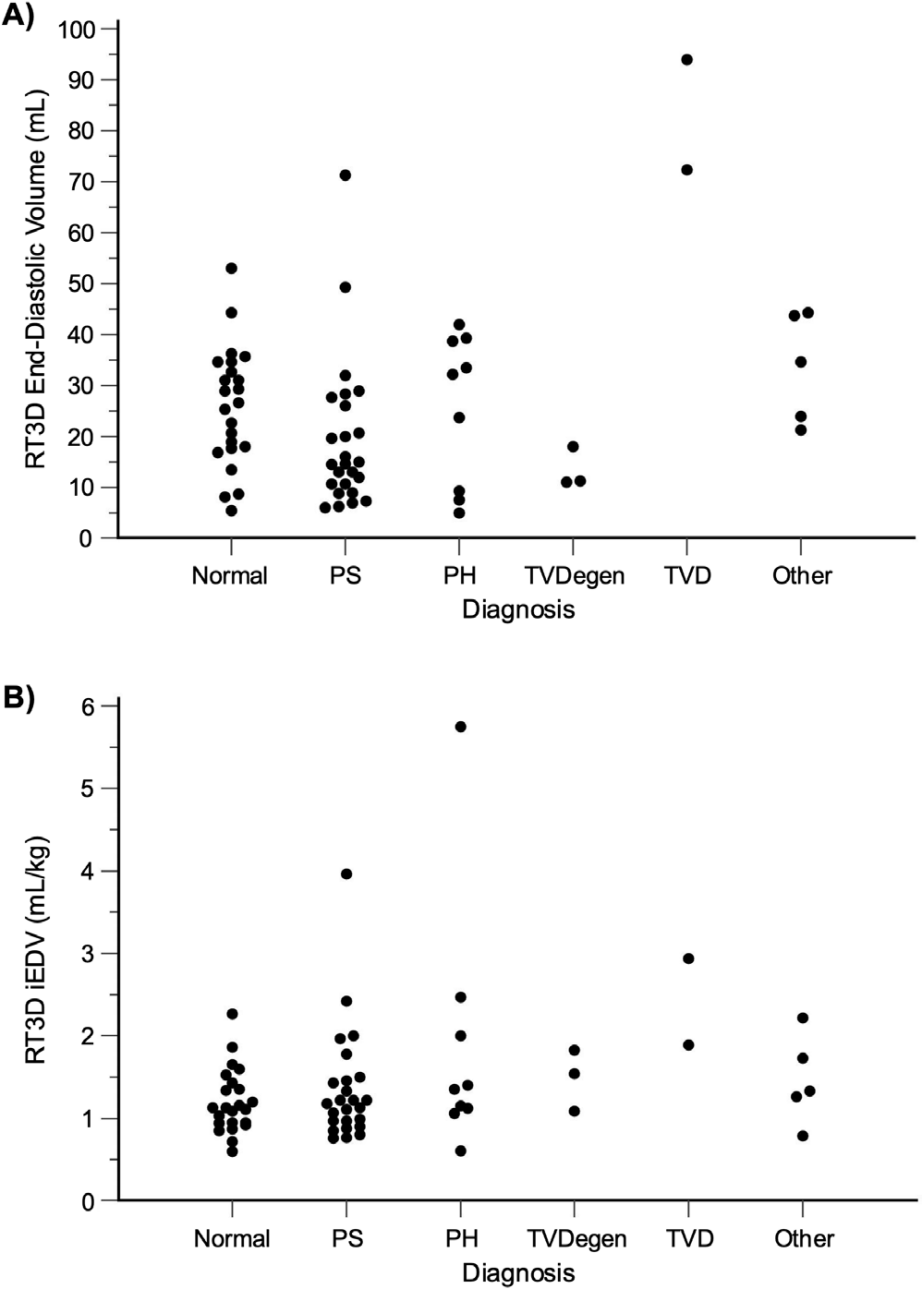
Distribution of RV end‐diastolic volumes by diagnosis. Dot plots show the distribution of absolute end‐diastolic volume (A) and iEDV (B) by diagnosis. The “other” category includes multiple congenital defects and one case of arrhythmogenic cardiomyopathy with dilation of the RV outflow tract. PH, pulmonary hypertension; PS, pulmonary valve stenosis; TVD, tricuspid valve dysplasia; TVDegen, tricuspid valve degeneration.

Echocardiography was performed on 72 dogs. However, five dogs were excluded because of an inability to complete RT3D measurements using the 4D auto RVQ module. All excluded dogs weighed < 6 kg, with four weighing < 3.5 kg. Consequently, the analysis was conducted on 67 dogs. However, the left apical two‐chamber view was missing in one dog, so methods involving the left apical two‐chamber view (AEM_RPS‐A2C_, LEM_RPS‐A2C_, AEM_A4C‐A2C_, and LEM_A4C‐A2C_) were analyzed in 66 dogs. Thirty‐three dogs (49%) were male and 34 (51%) were female. The median age was 4 (2–7) years, and the median weight was 18.0 (9.8–25.8) kg. Twenty‐eight breeds were represented, including mixed breed (21), French Bulldog (6), Labrador Retriever (5), Pit Bull Terrier (5), Australian Shepherd (3), Boxer (2), Chihuahua (2), German Shepherd (2), Shetland Sheepdog (2), and one each of Alaskan Klee Kai, Beagle, Bernese Mountain Dog, Bichon Frise, Border Collie, Cairn Terrier, Cavalier King Charles Spaniel, Dalmatian, English Springer Spaniel, Golden Retriever, Havanese, Italian Greyhound, Miniature Poodle, Miniature Pinscher, Miniature Schnauzer, Shih Tzu, Tibetan Terrier, Vizsla, and Whippet. Twenty‐three dogs had normal hearts, while 25 had pulmonary valve stenosis, nine had pulmonary hypertension, three had tricuspid valve degeneration, two had tricuspid valve dysplasia, four had multiple congenital defects (pulmonary valve stenosis with tricuspid valve dysplasia, pulmonary valve stenosis with cor triatriatum dexter, tricuspid valve dysplasia with cor triatriatum dexter, and tricuspid valve dysplasia with ventricular septal defect and pulmonary hypertension), and one had arrhythmogenic cardiomyopathy with RV outflow tract dilation. All median RV volumes, both absolute and body weight‐indexed, are shown in Table 1. The distribution of RT3D end‐diastolic volumes by diagnosis is provided in Figure 2. Notably, only a minority of dogs exhibited RT3D iEDVs exceeding the range observed in normal dogs.

**TABLE 1:** Median (interquartile range) of raw RV volumes and body weight‐indexed RV volumes of all echocardiographic methods.

Method	End‐diastolic volume (mL)	End‐systolic volume (mL)	iEDV (mL/kg)	iESV (mL/kg)
AEM_RPL‐A2C_	16.78 (10.43–29.36)	8.80 (4.38–13.97)	1.09 (0.81–1.43)	0.47 (0.39–0.75)
LEM_RPL‐A2C_	21.50 (13.35–37.48)	11.84 (6.37–21.39)	1.37 (1.10–1.72)	0.69 (0.57–0.92)
AEM_RPL‐RPS_	22.84 (13.38–33.80)	10.94 (5.45–17.03)	1.34 (1.04–1.81)	0.62 (0.45–0.92)
LEM_RPL‐RPS_	28.24 (17.14–43.06)	14.73 (7.79–25.23)	1.60 (1.34–2.22)	0.85 (0.65–1.18)
AEM_A4C‐A2C_	19.05 (9.84–30.05)	8.54 (4.65–16.27)	1.12 (0.87–1.40)	0.52 (0.37–0.68)
LEM_A4C‐A2C_	18.21 (10.69–28.09)	9.04 (5.39–15.82)	1.06 (0.91–1.33)	0.52 (0.41–0.72)
AEM_A4C‐RPS_	23.93 (14.21–37.15)	10.34 (5.54–19.59)	1.38 (1.07–1.79)	0.61 (0.46–0.86)
LEM_A4C‐RPS_	23.94 (13.61–34.50)	11.18 (6.33–19.48)	1.31 (1.09–1.70)	0.66 (0.50–0.89)
RT3D	21.33 (12.25–33.29)	10.00 (5.33–17.92)	1.20 (0.97–1.59)	0.60 (0.42–0.81)

The median of the differences and the concordance correlation coefficient for each AEM and LEM method compared to RT3D are detailed in Table S1.

**FIGURE 3:**
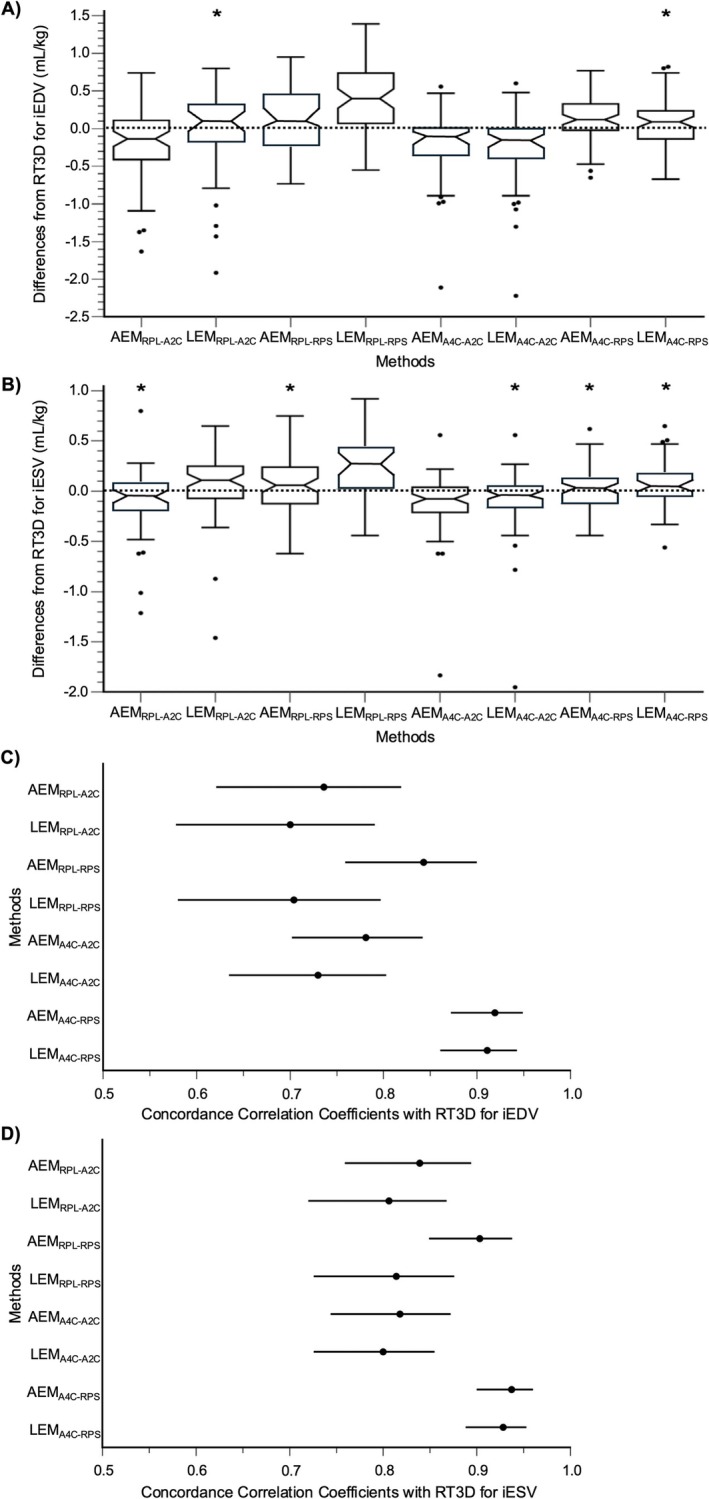
Bias and agreement of body weight‐indexed RV volumes for each AEM and LEM variant compared to RT3D. Notched box‐and‐whisker plots of the differences between each method and RT3D are shown for iEDV (A) and iESV (B). The notched areas outline the 95% confidence interval for the median of the differences. If this interval overlaps zero, then the corresponding method is considered to have no systematic bias with RT3D (marked by *). Additionally, forest plots of the concordance correlation coefficients and corresponding 95% confidence intervals between each method and RT3D are shown for iEDV (C) and iESV (D). Significant differences are determined when there is no overlap between intervals.

**FIGURE 4:**
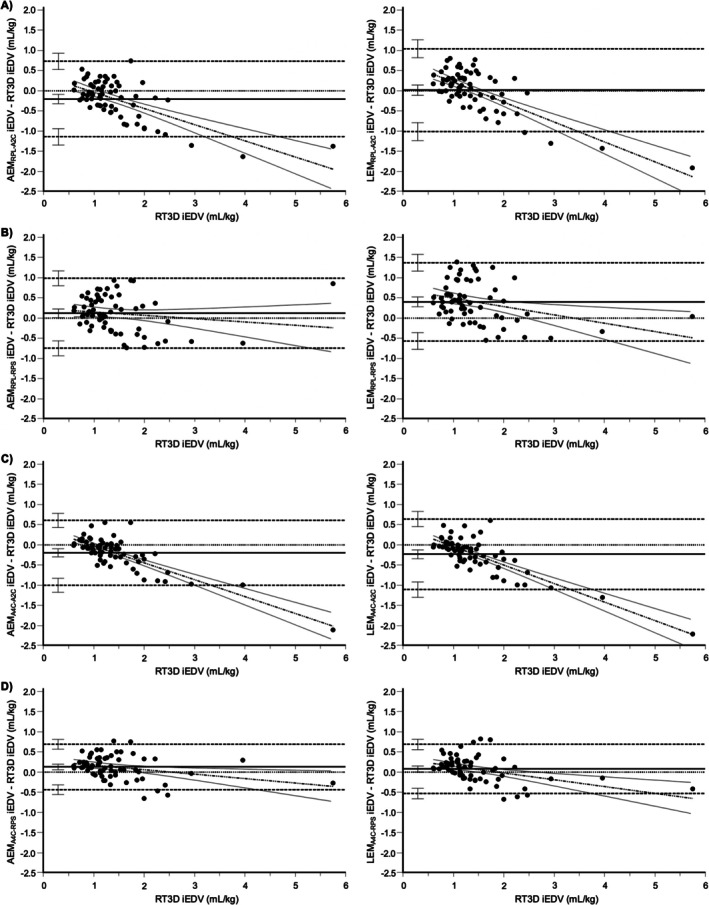
Bland–Altman plots comparing all AEM and LEM methods with RT3D for iEDV. The plots for AEM_RPL‐A2C_ (A), AEM_RPL‐RPS_ (B), AEM_A4C‐A2C_ (C), and AEM_A4C‐RPS_ (D) are displayed alongside their linear counterparts (LEM_RPL‐A2C_, LEM_RPL‐RPS_, LEM_A4C‐A2C_, and LEM_A4C‐RPS_, respectively). The line of equality (horizontal solid line), regression line of differences (alternating thick and thin dotted line), limits of agreement (horizontal thick dotted lines), and their 95% confidence intervals are included. Proportional bias is present when the 95% confidence interval for the slope of the regression line of differences does not include zero (i.e., does not include a flat line).

For iEDV, LEM_RPL‐A2C_, and LEM_A4C‐RPS_ had medians of the differences that narrowly included zero within their 95% confidence intervals, indicating the potential for no systematic bias (Figure 3A). The remaining methods using the perpendicular diameter from the left apical two‐chamber view (AEM_RPL‐A2C_, AEM_A4C‐A2C_, and LEM_A4C‐A2C_) underestimated RT3D, whereas those using the perpendicular diameter from the right parasternal short‐axis basilar view (AEM_RPL‐RPS_, LEM_RPL‐RPS_, and AEM_A4C‐RPS_) overestimated RT3D. In the Bland–Altman plots, only AEM_RPL‐RPS_ had a regression slope that was not significantly different from zero (*p* = 0.241; Figure 4). All other methods demonstrated greater negative bias at larger volumes, particularly those using the perpendicular diameter from the left apical two‐chamber view. This is consistent with positive proportional bias, in which the magnitude of bias (whether positive or negative) increases with increasing volume. Based on the concordance correlation coefficient, only AEM_A4C‐RPS_ and LEM_A4C‐RPS_ achieved at least moderate agreement with RT3D, performing significantly better than all other methods except AEM_RPL‐RPS_ (Figure 3C).

**FIGURE 5:**
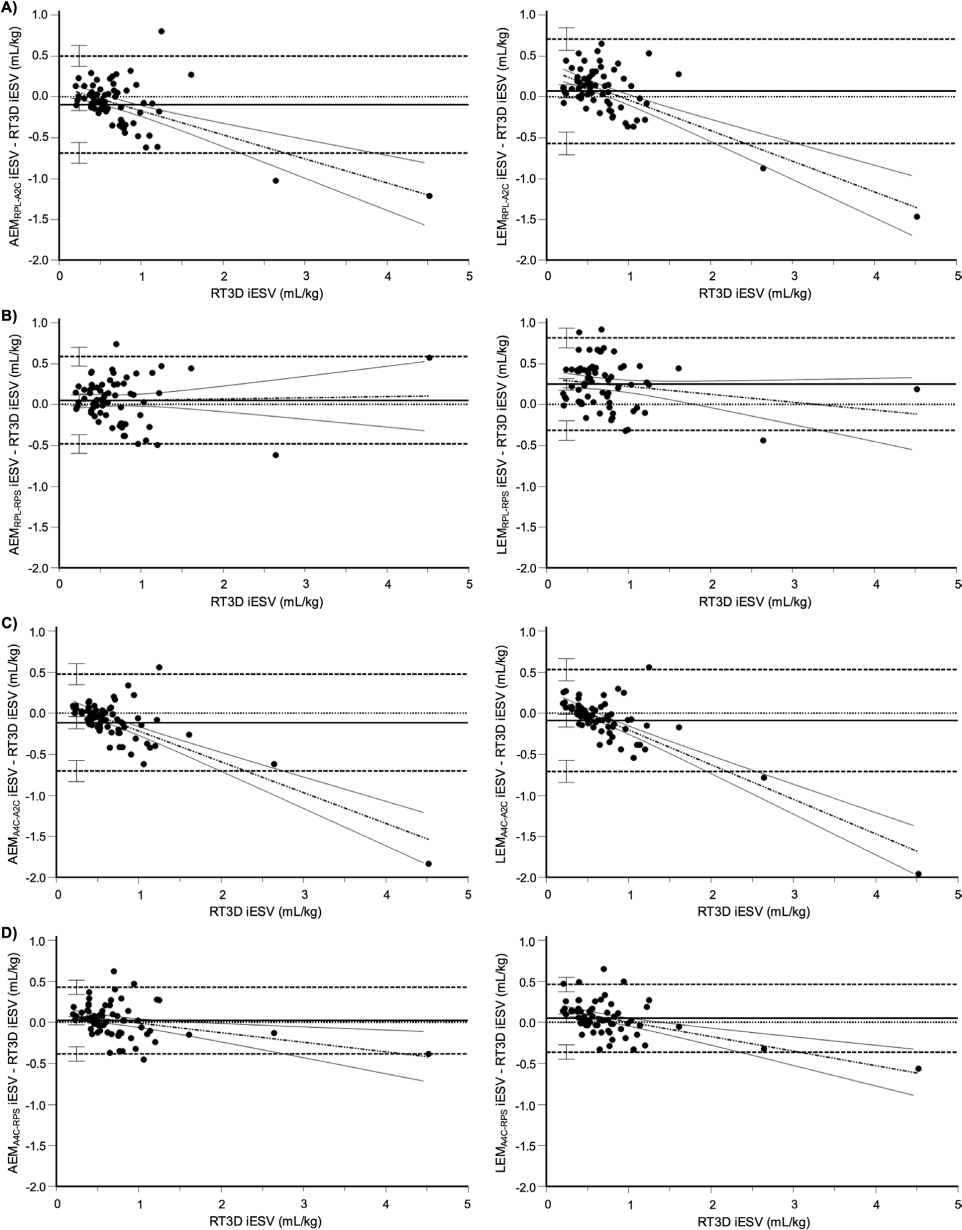
Bland–Altman plots comparing all AEM and LEM methods with RT3D for iESV. The plots for AEM_RPL‐A2C_ (A), AEM_RPL‐RPS_ (B), AEM_A4C‐A2C_ (C), and AEM_A4C‐RPS_ (D) are displayed alongside their linear counterparts (LEM_RPL‐A2C_, LEM_RPL‐RPS_, LEM_A4C‐A2C_, and LEM_A4C‐RPS_, respectively). The line of equality (horizontal solid line), regression line of differences (alternating thick and thin dotted line), limits of agreement (horizontal thick dotted lines), and their 95% confidence intervals are included. Proportional bias is present when the 95% confidence interval for the slope of the regression line of differences does not include zero (i.e., does not include a flat line).

For iESV, AEM_RPL‐A2C_, AEM_RPL‐RPS_, LEM_A4C‐A2C_, AEM_A4C‐RPS_, and LEM_A4C‐RPS_ had medians of the differences that included zero within their 95% confidence intervals (Figure 3B). Of the remaining methods, AEM_A4C‐A2C_ underestimated RT3D, while LEM_RPL‐A2C_ and LEM_RPL‐RPS_ overestimated RT3D. In the Bland–Altman plots, only AEM_RPL‐RPS_ (*p* = 0.806) and LEM_RPL‐RPS_ (*p* = 0.109) had regression slopes that were not significantly different from zero (Figure 5). All other methods demonstrated greater negative bias at larger volumes (i.e., positive proportional bias), again, particularly those using the perpendicular diameter from the left apical two‐chamber view. Based on the concordance correlation coefficient, AEM_RPL‐RPS_, AEM_A4C‐RPS_, and LEM_A4C‐RPS_ achieved at least moderate agreement with RT3D, with the latter two performing significantly better than the remaining methods (Figure 3D).

**FIGURE 6:**
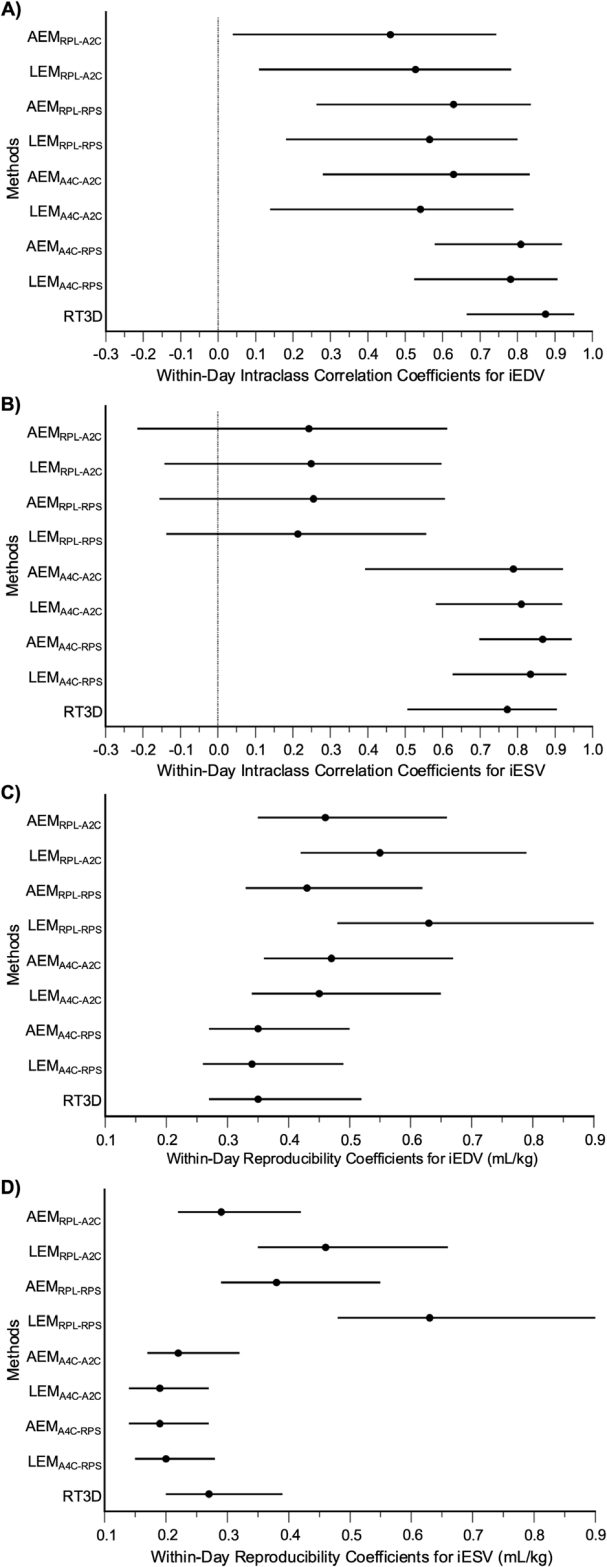
Within‐day intraclass correlation coefficients and reproducibility coefficients of all echocardiographic methods in healthy dogs. Forest plots of the intraclass correlation coefficients and corresponding 95% confidence intervals for each echocardiographic method are shown for iEDV (A) and iESV (B). Additionally, forest plots of the reproducibility coefficients and corresponding 95% confidence intervals for each echocardiographic method are shown for iEDV (C) and iESV (D). Significant differences are determined when there is no overlap between intervals.

Within‐day reproducibility was evaluated in 20 dogs with normal hearts, but RT3D reproducibility was analyzed in 19 dogs because of the inability to complete measurements in a 4.6‐kg Havanese dog using the 4D RVQ module. Five dogs (25%) were male and 15 (75%) were female. The median age was 5 (3–6) years, and the median weight was 22.7 (19.2–29.2) kg. Eleven breeds were represented, including mixed breed (8), German Shepherd (2), Labrador Retriever (2), and one each of Bernese Mountain Dog, Dalmatian, Golden Retriever, Havanese, Pit Bull Terrier, Shetland Sheepdog, Tibetan Terrier, and Vizsla. The coefficients of variation are presented in Table 2, and the intraclass correlation coefficients and reproducibility coefficients are detailed in Table S2. The iEDVs generally had coefficients of variation between 10% and 19% and moderate reproducibility based on intraclass correlation coefficients, except for AEM_RPL‐A2C_, which demonstrated poor reproducibility. However, none of the intraclass correlation coefficients or reproducibility coefficients differed significantly from one another (Figure 6A,C). The iESVs generally had coefficients of variation between 12% and 33%, with higher variability (> 25%) in methods using the right parasternal long‐axis four‐chamber view (AEM_RPL‐A2C_, LEM_RPL‐A2C_, AEM_RPL‐RPS_, LEM_RPL‐RPS_) and lower variability (< 16%) in those using the left apical four‐chamber view (AEM_A4C‐A2C_, LEM_A4C‐A2C_, AEM_A4C‐RPS_, LEM_A4C‐RPS_). Intraclass correlation coefficients similarly indicated poor reproducibility for the right parasternal long‐axis four‐chamber methods and moderate reproducibility for the left apical four‐chamber methods (Figure 6B). The lowest reproducibility coefficients were observed with LEM_A4C‐A2C_, AEM_A4C‐RPS_, and LEM_A4C‐RPS_, which were significantly lower than those of LEM_RPL‐A2C_, AEM_RPL‐RPS_, and LEM_RPL‐RPS_ (Figure 6D).

**TABLE 2:** Coefficients of variation of all echocardiographic methods.

	Coefficients of variation	
Method	iEDV	iESV
AEM_RPL‐A2C_	18.1	25.3
LEM_RPL‐A2C_	16.8	27.9
AEM_RPL‐RPS_	14.5	28.3
LEM_RPL‐RPS_	16.3	32.8
AEM_A4C‐A2C_	15.9	15.4
LEM_A4C‐A2C_	16.2	13.3
AEM_A4C‐RPS_	10.1	11.3
LEM_A4C‐RPS_	10.4	12.1
RT3D	11.5	17.4

## 4 Discussion

The AEM and LEM formulas provide 2D means to estimate RV volume, with several variants available. Among these, AEM_A4C‐RPS_ and LEM_A4C‐RPS_ best approximated RT3D while maintaining within‐day coefficients of variation below 13%. Both methods consistently demonstrated at least moderate agreement, significantly higher than that of the remaining methods except AEM_RPL‐RPS_. Aside from AEM_A4C‐RPS_ overestimating RT3D iEDV, neither method showed systematic bias relative to RT3D. Notably, although only a small number of dogs had large RV volumes, both methods exhibited negative bias at higher volumes. This proportional bias, however, was less pronounced than that seen with methods using the left apical two‐chamber view. For within‐day reproducibility, no significant differences were observed for iEDV across all methods. However, except for AEM_RPL‐A2C_, AEM_A4C‐RPS_ and LEM_A4C‐RPS_ showed significantly better reproducibility for iESV than the methods based on the right parasternal long‐axis four‐chamber view.

The AEM and LEM formulas are based on a quarter‐ellipsoid shape [14]. The volume of a quarter‐ellipsoid is:

$$\text{Volume}=\frac{1}{4}\times \frac{4}{3}\pi \times r_{1}\times r_{2}\times r_{3}=\frac{\pi }{3}\times r_{1}\times r_{2}\times r_{3}$$

where *r*_1_, *r*_2_, and *r*_3_ represent the radii along the three perpendicular axes. Starting from the first unsimplified formula, substituting in RV area as the area of a quarter‐ellipse ($\mathrm{RV}\;\text{area}=\frac{\pi \times r_{1}\times r_{2}}{4}$) and the perpendicular diameter as twice the remaining radius ($\text{perpendicular diameter}=2\times r_{3}$) gives [14, 16]:

$$\begin{array}{c} \text{Volume}=\frac{2}{3}\times \left(\frac{\pi \times r_{1}\times r_{2}}{4}\right)\times \left(2\times r_{3}\right)\\ \;=\frac{2}{3}\times \left(\text{RVarea}\right)\times \left(\text{perpendicular diameter}\right) \end{array}$$

Starting from the simplified quarter‐ellipsoid formula, substituting the RV basilar width for *r*_1_, the RV length for *r*_2_, and the same perpendicular diameter gives [16]:

$$\begin{array}{c} \text{Volume}=\frac{\pi }{3}\times r_{1}\times r_{2}\times \frac{\text{perpedicular diameter}}{2}\\ \;=\frac{\pi }{6}\times \left(\mathrm{RV}\;\text{basilar width}\right)\times \left(\mathrm{RV}\;\text{length}\right)\\ \;\times \left(\text{perpendicular diameter}\right) \end{array}$$

The LEM methods were also investigated because they offer several advantages over the traditional AEM methods. First, linear measurements are quick and efficient to perform. Second, they do not require visualization of the RV free wall, which can be challenging to obtain due to lung artifacts [16]. In any case, both AEM and LEM methods have shown good correlation with other modalities in humans, including MRI and thermodilution (for stroke volume assessment) [14, 15, 16, 27]. However, perfect agreement remains elusive, as these methods often underestimated or overestimated volumes compared to MRI. Furthermore, as in this study, limits of agreement were wide, approaching 20%–30% of the measured volume [14, 27]. These findings suggest that caution is warranted when interpreting ellipsoid model calculations as definitive volumes. Nevertheless, the AEM and LEM methods are appealing because of their simplicity and reasonable approximation of the RV shape.

A variety of ellipsoid model variants were investigated in this study, partly due to the lack of uniform measurement methodologies and partly to explore the utility of alternate views. The first major distinction among the ellipsoid model variants was the imaging view used to measure RV parameters (area, basilar width, and length), which could be obtained from either the right parasternal long‐axis or left apical four‐chamber views. In all human studies, these parameters were obtained from the left apical four‐chamber view focused on the RV [14, 15, 16], consistent with recommendations from the American Society of Echocardiography [20]. We incorporated right parasternal long‐axis RV measurements because of their ease of acquisition, their use as the basis for most subjective assessments, and the availability of established quantitative right heart ratios [9]. However, LEM_RPL‐RPS_ exhibited significantly greater systematic bias and lower agreement than AEM_A4C‐RPS_ and LEM_A4C‐RPS_ for both iEDV and iESV, although AEM_RPL‐RPS_ did not. Based on our experience, this discrepancy may be attributed to greater beat‐to‐beat variation in the right parasternal long‐axis view, particularly at end‐systole when distinguishing the RV cavity from trabeculated myocardium and papillary muscles becomes more challenging. In contrast, the left apical view more consistently opens the RV cavity for clearer delineation throughout the cardiac cycle, and positioning the transducer at the apex may help minimize the impact of cardiac motion. This may also explain why AEM_RPL‐RPS_ and LEM_RPL‐RPS_ demonstrated significantly poorer within‐day reproducibility for iESV, as reflected by markedly high coefficients of variation (> 25%), lower intraclass correlation coefficients, and higher reproducibility coefficients compared to AEM_A4C‐RPS_ and LEM_A4C‐RPS_. Another explanation could be that RT3D is also acquired from the left apical view, which may inherently favor agreement with left apical‐based methods. In conclusion, acquiring RV parameters from the left apical four‐chamber view may be preferable, as methods using the right parasternal long‐axis four‐chamber view showed poor agreement and reproducibility, which may limit their clinical utility.

Notably, basilar width was measured not at the annular level but at the widest portion of the basilar one‐third of the RV [16]. Although this does not directly represent the radius of an ellipsoid, it is the maximal short‐axis dimension that the radius is intended to reflect, and is therefore the recommended measurement [19, 28]. To enhance comparability with human studies, we adopted the same approach.

The second major distinction among the ellipsoid model variants was the location from which the perpendicular diameter was measured, which may be either the left ventricle [16] or the right ventricle [14, 15]. While using the left ventricle produced favorable results in humans, it introduced significant bias in dogs, particularly at larger volumes. This discrepancy likely arises because chamber enlargement is more pronounced in veterinary patients, as interventional or surgical corrections, if available, are typically implemented earlier in humans to prevent severe dilation. When the right ventricle undergoes severe enlargement while the left ventricle remains unchanged, the resulting mismatch in chamber dimensions leads to substantial proportional bias. Therefore, deriving the perpendicular diameter from the right ventricle appears to be a more appropriate choice for ellipsoid model calculations in dogs.

Although certain ellipsoid model variants showed promise in approximating RT3D volumes, this study included only a few dogs with large RV volumes, which strongly influenced the degree of proportional bias. This reflects our hospital's caseload, where pulmonary valve stenosis—typically characterized by smaller chamber volumes—is the most common presenting right‐sided heart disease. Exceptionally large RV volumes were primarily observed with pulmonary hypertension secondary to caval syndrome (on recheck following heartworm retrieval), pulmonary valve stenosis with severe myocardial failure, and tricuspid valve dysplasia, but few such dogs were enrolled. Although all enrolled dogs were required to have normal left heart dimensions, no inclusion or exclusion criteria were applied based on RV chamber size, primarily to avoid excluding dogs with pulmonary valve stenosis that would likely have normal chamber dimensions. Consequently, dogs with mild right‐sided disease were not excluded, further contributing to the predominance of normal or near‐normal RV volumes. A longer enrollment period with stratified recruitment would be necessary to achieve a more balanced distribution of RV volumes, which could alter the observed systematic and proportional biases. Additionally, we opted not to exclude any outliers, as important trends emerged that are likely valid. As discussed, the strong proportional bias allowed us to exclude the clinical utility of the ellipsoid model variants using left ventricular‐derived perpendicular diameters. Among the remaining methods, only AEM_RPL‐RPS_ exhibited no proportional bias for both iEDV and iESV. This suggests that AEM and LEM methods in general may be less reliable at larger volumes, potentially due to geometric shape changes—a limitation not observed in human studies, where markedly enlarged RV volumes are rare. However, the small sample size at the upper end of the volume range still limits definitive conclusions about the accuracy of AEM_RPL‐RPS_, underscoring the need for refined recruitment strategies.

The study initially aimed to assess interoperator reproducibility, but all agreement measurements were ultimately performed jointly by a board‐certified cardiologist and a cardiology resident‐in‐training. Instead of the standard left apical four‐chamber view that emphasizes the left ventricle, a “RV‐focused view” is recommended for all RV‐related measurements. However, the absence of fixed anatomic landmarks increases the likelihood for variations in cut planes that can lead to different dimensions, which posed a challenge given the resident's limited imaging experience at the time [20]. To mitigate this, the two‐operator setup enabled cross‐checking to ensure appropriate image selection, maximizing visualization of the RV cavity while avoiding foreshortening and inclusion of the aorta or caudal vena cava. While this approach likely improved image quality and measurement consistency, it may have artificially enhanced agreement and reproducibility, thereby limiting the generalizability of the results to single‐operator settings. Additionally, within‐day reproducibility was assessed only by the cardiologist due to residency duration constraints and was limited to healthy dogs owned by students and staff. Performing a second echocardiogram required three additional hours of waiting time, which was logistically challenging to accommodate for clinical patients. These factors likely contributed to better reproducibility than might be expected with different operators.

Although MRI is considered the gold standard for volumetric quantification in human medicine, RT3D was chosen as the reference standard in this study. Because RT3D is performed similarly to routine echocardiography and does not require anesthesia, it offered a practical means of acquiring a large sample size with minimal risk [29]. However, human studies have shown that RT3D typically underestimates MRI‐derived RV volumes despite strong correlations [7, 21, 30, 31, 32], a pattern also observed with left ventricular volumes [32, 33, 34]. The same finding has also been reported in dogs [11]. This underestimation is likely due to the lower spatial resolution of RT3D, as MRI's high in‐plane resolution allows for endocardial tracings to extend wider. Additionally, there is an inherent tradeoff between spatial and temporal resolution; increasing temporal resolution reduces spatial resolution, which may contribute to lower volume estimates [31, 33]. To balance these factors, we selected a frame rate slightly above 30 frames per second, though this can still be problematic in tachycardic dogs. Further limitations stem from the 4D Auto RVQ module, which can only be applied to images acquired using the low‐frequency 4Vc‐4D transducer. This constraint results in suboptimal resolution and measurement difficulties in smaller dogs and may preclude data acquisition in those weighing less than 6 kg. While these limitations must be acknowledged, restricted access to gold standard imaging modalities remains a common challenge in veterinary echocardiographic research.

Beyond RV volume distribution, operator roles, and choice of reference standard, this study had several other limitations. First, heart rates were not standardized. Second, the measurements varied by three to five cycles at the discretion of the operator. However, this was elected as it mimicked the authors' current clinical practice. Third, RT3D volume was calculated via a semi‐automated module specific to the GE system, so results may differ with other ultrasound systems and software. Fourth, recruitment was limited to a two‐year time frame (constrained by residency duration) and the lack of sedation restricted sample size. While additional operators could have expanded recruitment, no other clinician routinely used or was trained in these modalities. Fifth, the study involved only two operators from the same institution, and the resident was trained by the cardiologist. These factors likely artificially lowered variability. Ideally, different operators from different institutions would be included, but availability of the same 4D Auto RVQ module is limited. Finally, ejection phase indices, such as ejection fraction, were not evaluated, as the primary objective was to assess bias and agreement in geometric modeling of volume. Inaccuracies and variability in end‐diastolic and end‐systolic volumes could be compounded in the calculation of ejection fraction.

In summary, AEM_A4C‐RPS_ and LEM_A4C‐RPS_ were the 2D echocardiographic variants that most closely approximated RT3D‐derived RV volume while exhibiting higher within‐day reproducibility for iESV. These methods, by virtue of their simplicity and ease of use, offer potential alternatives to the more complex RT3D, which requires specialized equipment and more extensive training. However, further research is necessary to assess their performance across a broader range of RV volumes and with multiple operators to ensure reliability in diverse clinical settings. Additionally, the impact of these methods on patient outcomes should be explored to determine their broader applicability in veterinary cardiology.

## Disclosure

Authors declare no off‐label use of antimicrobials.

## Ethics Statement

Approved by the Cornell University Institutional Animal Care and Use Committee, protocol # 2022‐0204. Authors declare human ethics approval was not needed.

## Conflicts of Interest

The authors declare no conflicts of interest.

## Supporting information


**Supplemental Figure S1:** Multi‐beat acquisition of RT3D images. The three left apical long‐axis views are visible along the left side of the figure. Each line (solid and dotted) in these views indicates the level corresponding to the short‐axis views. The top short‐axis views correspond to the most apical solid line, the middle short‐axis views to the middle dotted line, and the bottom short‐axis views to the most basilar solid line. The crescentic shape of the RV is evident in the short‐axis views, though the anterior aspect is obscured by lung artifact.

**Data S1:** Tables.

**Video S1:** Supplemental Video Measurement of RT3D volume using the 4D Auto RVQ module. First, a vertical axis was manually aligned through the center of the tricuspid valve and the RV apex. Next, the tricuspid annulus (free wall and septal aspects) and RV apex were manually marked on the long‐axis view, while the anterior, lateral, and posterior landmarks were identified on the short‐axis view. The software then generated an automated endocardial border trace, presented in a 3 × 3 multislice layout: the first column allowed navigation through the full cardiac cycle, the second displayed only end‐diastolic frames, and the third showed only end‐systolic frames. The multislice model was carefully reviewed in all planes to verify the accuracy of border detection, with manual refinement made where necessary. Finally, a time–volume curve was produced, along with calculated values for RV volume and functional parameters.
