# Supplementary figures and images for: Comparing Two‐Dimensional Ellipsoid Model Variants in Estimating Three‐Dimensional Echocardiographic Right Ventricular Volume in Dogs
Source: J Vet Intern Med. 2025 Aug 27;39(5):e70232. doi: 10.1111/jvim.70232 (PMC12385342; doi:10.1111/jvim.70232)

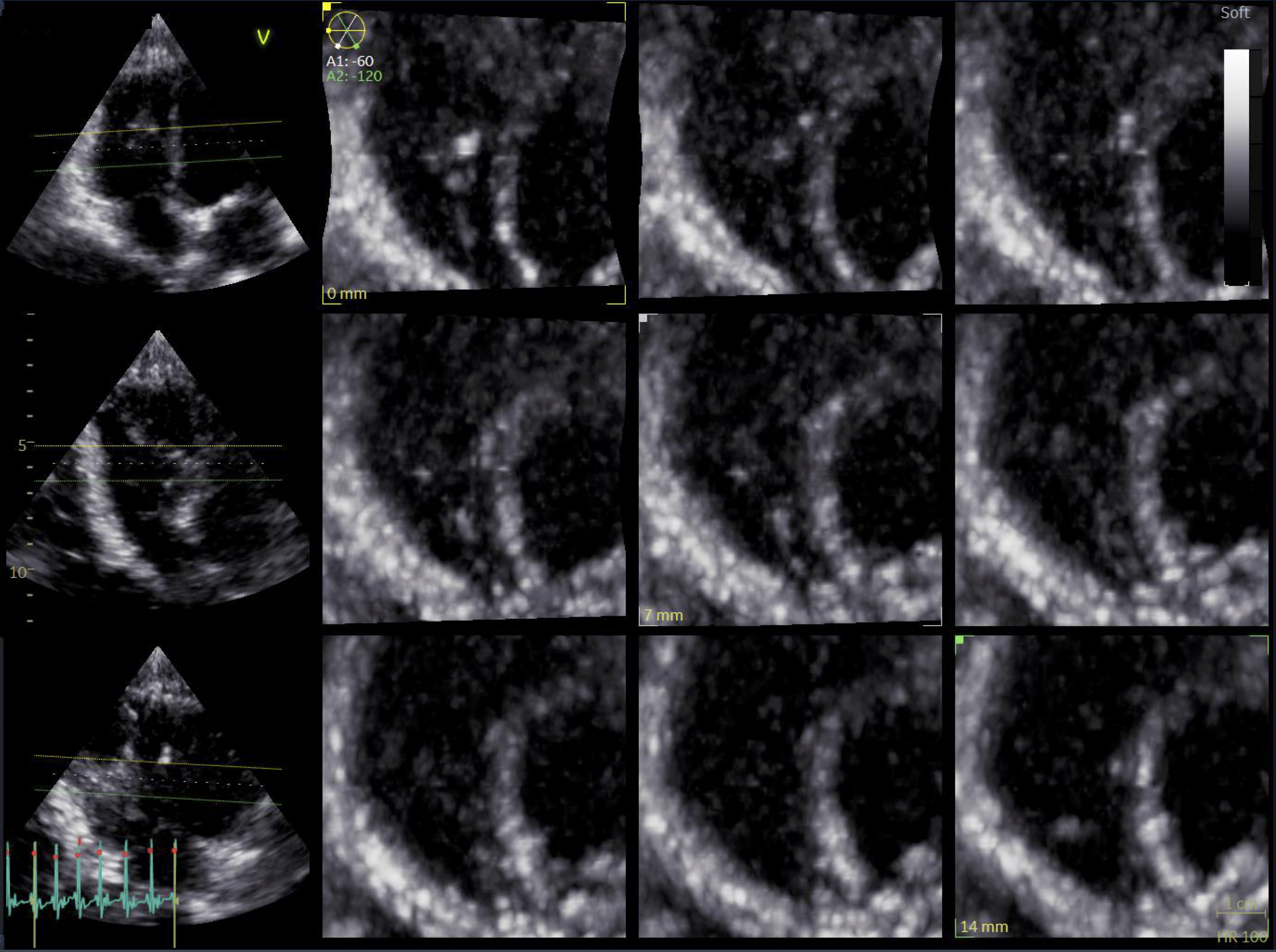

Supplement: Supplementary file 1 — Supplemental Figure S1: Multi‐beat acquisition of RT3D images. The three left apical long‐axis views are visible along the left side of the figure. Each line (solid and dotted) in these views indicates the level corresponding to the short‐axis views. The top short‐axis views correspond to the most apical solid line, the middle short‐axis views to the middle dotted line, and the bottom short‐axis views to the most basilar solid line. The crescentic shape of the RV is evident in the short‐axis views, though the anterior aspect is obscured by lung artifact. [file JVIM-39-e70232-s001.tiff]
