# Supplementary material for: Comparing Two‐Dimensional Ellipsoid Model Variants in Estimating Three‐Dimensional Echocardiographic Right Ventricular Volume in Dogs
Source: J Vet Intern Med. 2025 Aug 27;39(5):e70232. doi: 10.1111/jvim.70232 (PMC12385342; doi:10.1111/jvim.70232)
Supplement: Supplementary file 2 — Data S1: Tables. [file JVIM-39-e70232-s003.docx]

**Supplemental Table I - Bias and agreement of body weight-indexed RV volumes for each AEM and LEM variant compared to RT3D.**

|  | **iEDV** | | | | | **iESV** | | | | |
| --- | --- | --- | --- | --- | --- | --- | --- | --- | --- | --- |
|  | **Differences (mL/kg)** | | **Concordance Correlation** | | | **Differences (mL/kg)** | | **Concordance Correlation** | | |
| **Method** | **Median**  **(95% CI)** | **Interquartile range** | **r_c_**  **(95% CI)** | **r_p_** | **C_b_** | **Median**  **(95% CI)** | **Interquartile range** | **r_c_**  **(95% CI)** | **r_p_** | **C_b_** |
| **AEM_RPL-A2C_** | -0.14  (-0.22 – -0.05) | -0.41 – 0.12 | 0.736  (0.621 – 0.819)^a^ | 0.802 | 0.917 | -0.07  (-0.12 – 0.00) | -0.19 – 0.08 | 0.839  (0.759 – 0.894)^a^ | 0.870 | 0.965 |
| **LEM_RPL-A2C_** | 0.11  (-0.04 – 0.23) | -0.17 – 0.32 | 0.700  (0.578 – 0.791)^b^ | 0.756 | 0.926 | 0.11  (0.03 – 0.17) | -0.07 – 0.25 | 0.806  (0.720 – 0.868)^b^ | 0.853 | 0.946 |
| **AEM_RPL-RPS_** | 0.09  (0.04 – 0.28) | -0.22 – 0.46 | 0.843  (0.759 – 0.900) | 0.855 | 0.986 | 0.06  (-0.01 – 0.15) | -0.13 – 0.24 | 0.903  (0.849 – 0.938) | 0.911 | 0.990 |
| **LEM_RPL-RPS_** | 0.40  (0.27 – 0.48) | 0.07 – 0.74 | 0.704  (0.580 – 0.797)^c^ | 0.801 | 0.879 | 0.28  (0.14 – 0.40) | 0.03 – 0.43 | 0.814  (0.726 – 0.876)^c^ | 0.886 | 0.919 |
| **AEM_A4C-A2C_** | -0.10  (-0.22 – -0.05) | -0.35 – 0.02 | 0.781  (0.702 – 0.842)^d^ | 0.899 | 0.870 | -0.08  (-0.12 – -0.01) | -0.21 – 0.04 | 0.818  (0.744 – 0.872)^d^ | 0.896 | 0.913 |
| **LEM_A4C-A2C_** | -0.15  (-0.27 – -0.08) | -0.39 – 0.00 | 0.730  (0.635 – 0.803)^e^ | 0.867 | 0.841 | -0.06  (-0.09 – 0.00) | -0.16 – 0.06 | 0.800  (0.726 – 0.855)^e^ | 0.890 | 0.898 |
| **AEM_A4C-RPS_** | 0.12  (0.06 – 0.19) | -0.02 – 0.33 | 0.919  (0.872 – 0.949)^a,b,c,d,e^ | 0.932 | 0.986 | 0.03  (-0.02 – 0.08) | -0.12 – 0.14 | 0.937  (0.900 – 0.960)^a,b,c,d,e^ | 0.939 | 0.997 |
| **LEM_A4C-RPS_** | 0.09  (0.00 – 0.13) | -0.14 – 0.25 | 0.911  (0.861 – 0.943)^a,b,c,d,e^ | 0.920 | 0.990 | 0.04  (-0.01 – 0.12) | -0.04 – 0.17 | 0.928  (0.888 – 0.953)^b,c,d,e^ | 0.939 | 0.987 |
| Abbreviations: C_b_, bias correction factor; CI, confidence intervals, r_c_, concordance correlation coefficient; r_p_, Pearson correlation coefficient  ^a^Significantly different from AEM_RPL-A2C_  ^b^Significantly different from LEM_RPL-A2C_  ^c^Significantly different from LEM_RPL-RPS_  ^d^Significantly different from AEM_A4C-A2C_  ^e^Significantly different from LEM_A4C-A2C_ | | | | | | | | | | |

**Supplemental Table 2 - Within-day intraclass correlation coefficients and reproducibility coefficients of all echocardiographic methods in healthy dogs.**

|  | **iEDV** | | **iESV** | |
| --- | --- | --- | --- | --- |
| **Method** | **ICC**  **(95% CI)** | **RC (mL/kg)**  **(95% CI)** | **ICC**  **(95% CI)** | **RC (mL/kg)**  **(95% CI)** |
| **AEM_RPL-A2C_** | 0.460  (0.039 – 0.744) | 0.46  (0.35 – 0.66) | 0.243  (-0.215 – 0.613)^a^ | 0.29  (0.22 – 0.42)^a^ |
| **LEM_RPL-A2C_** | 0.527  (0.110 – 0.783) | 0.55  (0.42 – 0.79) | 0.249  (-0.142 – 0.597)^b^ | 0.46  (0.35 – 0.66)^b^ |
| **AEM_RPL-RPS_** | 0.629  (0.263 – 0.836) | 0.43  (0.33 – 0.62) | 0.255  (-0.156 – 0.607)^c^ | 0.38  (0.29 – 0.55)^c^ |
| **LEM_RPL-RPS_** | 0.565  (0.182 – 0.801) | 0.63  (0.48 – 0.90) | 0.213  (-0.137 – 0.557)^d^ | 0.63  (0.48 – 0.90)^a,d^ |
| **AEM_A4C-A2C_** | 0.629 (0.280 – 0.833) | 0.47  (0.36 – 0.67) | 0.789  (0.393 – 0.922) | 0.22  (0.17 – 0.32)^b,d^ |
| **LEM_A4C-A2C_** | 0.541  (0.139 – 0.790) | 0.45  (0.34 – 0.65) | 0.810  (0.582 – 0.920)^d^ | 0.19  (0.14 – 0.27)^b,c,d^ |
| **AEM_A4C-RPS_** | 0.809  (0.579 – 0.920) | 0.35  (0.27 – 0.50) | 0.867  (0.698 – 0.945)^a,b,c,d^ | 0.19  (0.14 – 0.27)^b,c,d^ |
| **LEM_A4C-RPS_** | 0.781  (0.524 – 0.908) | 0.34  (0.26 – 0.49) | 0.834  (0.627 – 0.931)^a,b,c,d^ | 0.20  (0.15 – 0.28)^b,c,d^ |
| **RT3D** | 0.875  (0.665 – 0.952) | 0.35  (0.27 – 0.52) | 0.772  (0.506 – 0.905) | 0.27  (0.20 – 0.39)^d^ |
| Abbreviations: CI, confidence intervals; ICC, intraclass correlation coefficient; RC, reproducibility coefficient  ^a^Significantly different from AEM_RPL-A2C_  ^b^Significantly different from LEM_RPL-A2C_  ^c^Significantly different from AEM_RPL-RPS_  ^d^Significantly different from LEM_RPL-RPS_ | | | | |
